# Oral administration of chestnut tannins to reduce the duration of neonatal calf diarrhea

**DOI:** 10.1186/s12917-018-1549-2

**Published:** 2018-07-28

**Authors:** F. Bonelli, L. Turini, G. Sarri, A. Serra, A. Buccioni, M. Mele

**Affiliations:** 1 Dipartimento di Scienze Veterinarie, via Livornese snc, Università di Pisa, via Livornese, 56122, San Piero a Grado, Pisa, Italy; 2 Centro di Ricerche Agro-ambientali “E. Avanzi” – Università di Pisa, via Vecchia di Marina, 6, 56122, San Piero a Grado, Pisa, Italy; 3 Dipartimento di Scienze delle Produzioni Agroalimentari e Ambientali, Università di Firenze, via delle Cascine, 5, 50100 Florence, Italy

**Keywords:** Calf, Neonatal diarrhea, Chestnut tannins, Phytotherapy

## Abstract

**Background:**

Neonatal calf diarrhea is generally caused by infectious agents and is a very common disease in bovine practice, leading to substantial economic losses. Tannins are known for their astringent and anti-inflammatory properties in the gastro-enteric tract. The aim of this study was to evaluate the effect of the oral administration of chestnut tannins (*Castanea sativa* Mill.) in order to reduce the duration of calf neonatal diarrhea. Twenty-four Italian Friesian calves affected by neonatal diarrhea were included. The duration of the diarrheic episode (DDE) was recorded and the animals were divided into a control group (C), which received Effydral® in 2 l of warm water, and a tannin-treated group (T), which received Effydral® in 2 l of warm water plus 10 g of extract of chestnut tannins powder. A Mann-Whitney test was performed to verify differences for the DDE values between the two groups.

**Results:**

The DDE was significantly higher in group C than in group T (*p* = 0.02), resulting in 10.1 ± 3.2 and 6.6 ± 3.8 days, respectively.

**Conclusions:**

Phytotherapic treatments for various diseases have become more common both in human and in veterinary medicine, in order to reduce the presence of antibiotic molecules in the food chain and in the environment. Administration of tannins in calves with diarrhea seemed to shorten the DDE in T by almost 4 days compared to C, suggesting an effective astringent action of chestnut tannins in the calf, as already reported in humans. The use of chestnut tannins in calves could represent an effective, low-impact treatment for neonatal diarrhea.

## Background

Calf diarrhea is a very common disease in bovine practice, and is generally caused by infectious agents [1]. The most common pathogens involved are *Rotavirus*, *Coronavirus*, *Cryptosporidium parvum* and *Escherichia coli*, especially in animals less than one month old [1, 2]. Calf diarrhea causes substantial economic losses in the dairy industry, due treatment costs, a decreased growth rate in the calves, and a higher replacement rate due to culling or death [2].

In order to reduce the presence of antibiotic molecules in the food chain and in the environment, phytotherapic treatments are now commonly used both in human [3] and in veterinary medicine. Various formulations of polyphenols, such as bacteriostatic, anti-clostridial and astringent, have been tested, especially in monogastric nutrition [4–7]. Some phytotherapic options have been evaluated for gastrointestinal diseases, such as gastric ulcers and gastric lesions, in rats, piglets, horses, and calves [8–10]. Pomegranate-residue supplements have been used in neonatal calves affected by *Cryptosporidium parvum*, resulting in a reduction in the fecal oocyst count, as well as in intensity and duration of diarrhea [11].

Tannins are a complex group of polyphenolic compounds which are present in several plants as secondary metabolites against pathogens [12]. In ruminants, in vitro and in vivo trials have demonstrated that tannins can improve animal performance and reduce the impact of gastrointestinal parasitism, nitrogen pollution, and methane emission from rumen fermentation [13, 14]. Tannins may interfere with digestive processes by binding dietary protein, by modulating the activity of rumen micro-organisms, and by reducing the growth of the bacterial population [15–17].

In ruminants, tannins tend to decrease the rate of protein degradation in the rumen [18]. Tannins, in fact, inhibit the growth of proteolytic bacteria and form tannin–protein complexes in the rumen. This effect may significantly vary according to the chemical structure of the tannin and to the amount of tannin included in the diet. However, the reduction of protein degradation in the rumen increases the total amount of protein digested in the duodenum, because the tannin-protein complexes are dissociated in the abomasum, releasing protein. As a consequence, the use of moderate doses of tannins in the diet of ruminants usually improves animal performance [18, 19].

The astringent and anti-inflammatory effects of tannins on the gastro-enteric tract have been demonstrated in avian [5, 20] and swine species [9], and the effects of tannins on liver function have been evaluated in newborn calves [21].

The aim of the present study was to evaluate the effect of oral administration of chestnut tannins (*Castanea sativa*) in the treatment of calf neonatal diarrhea.

## Methods

This in vivo blinded study was approved by the Institutional Animal Care and Use Committee (OBA, Pisa, prot. n. 33,479/2016 of 29.06.2016) and was carried out at the University of Pisa’s dairy farm, where nearly 100 animals are maintained in free-stall conditions.

During the study period, the population consisted of 40 Italian Friesian calves aged between 1 and 60 days. All the calves underwent the same management condition. Briefly, immediately after birth they were weighed and then housed in a single straw-bedding pen (2.5 × 2 m) that leads contact between each other. Two L of good colostrum (≥50 g/L of Ig) from their own dam, or from the colostrum bank, were administered as soon as the calf could drink (30 min – 2 h). Another 2 L were administered within the next 4–8 h in order to achieve a good passive transfer immunity [22]. All the calves received a total of 4 L of colostrum, twice a day, until the third day of life. Then, they received 6 L of whole milk at 39 °C, twice a day until the third week of life.

All the feeding procedures were conducted by an expert operator and by using a nipple bucket. Even when there were cases of diarrhea, there were still no feeding restrictions or changes of feeding regime. From the third day of life, fresh and clean water were provided to each calf ad libitum. Free choice-hay was administered after the first week of life. The calves were weighed and moved into a collective pen after the third week of life.

The inclusion criteria were calves up to three weeks of life and the manifestation of diarrhea, defined as a fecal score ≥ 1 [1]. Briefly, the fecal score represents the evaluation of feces fluidity as reported in literature: score 0 means “normal”, thus firm, but not hard and the original form is distorted slightly after dropping to the floor and settling; score 1 means “soft”, thus does not hold its form, but piles and spreads slightly (like soft-serve ice cream); score 2 means “runny”, thus spreads readily to about 6 mm depth. (i.e., pancake batter); score 3 means “watery”, thus liquid consistency, splatters. (i.e., orange juice) [23]. Since the first day of diarrhea (T0), the fecal score (FS) was recorded daily by the same expert operator until the complete recovery from diarrhea, at the same time, the physical examination has been done. The duration of a diarrheic episode (DDE) was defined as the period (in days) between the first diarrhea outbreak (fecal score ≥ 1) and the normalizing of the FS (fecal score = 0). Once the diarrhea had started, a fecal sample was collected from each calf by manual restraint. A gloved, lubricated finger was passed gently through the anus in order to massage the rectal wall and to stimulate rectal evacuation. Fresh feces were then collected in two different sterile tubes: one aliquot was immediately tested with a rapid ELISA test (test strips for detection of *Rotavirus*, *Coronavirus*, *E. coli* F5 and *C. parvum* in bovine feces, Biox Diagnostics, Belgium), while the second aliquot was stored in a refrigerated bag and evaluated within one hour for gastrointestinal parasites, according to Izzo et al. (2015) [1].

At the inclusion time, calves were randomly assigned to a control group (C) or a tannin-treated group (T), both made up of 12 calves (6 males and 6 females in each of the two groups). All the calves enrolled in group C received Effydral® (Italy Zoetis Ltd.) (sodium chloride 2.34 g, potassium chloride 1.12 g, sodium bicarbonate 6.72 g, citric acid anhydrous 3.84 g, lactose monohydrate 32.44 g, glycine 2.25 g) in 2 L of warm water q24h. The calves assigned to group T received Effydral® (Italy Zoetis Ltd.) in 2 L of warm water plus 10 g of chestnut tannins as extract powder (750 g/kg of dry matter equivalent of tannic acid; Mauro Saviola Group srl, Radicofani, Siena, Italy) q24h. The chemical composition of the powder is described in Campo et al. (2016) [24]. The powder adopted in the present study was produced in a single batch and analyzed by the manufacturer at the beginning of the study. Both solutions were administered using a graduated calves bottle in order to ensure the intake of the entire quantity. The bottle was equipped with a flexible rubber nipple (10 cm of length) specific for calf feeding.

Calves in both groups received the solutions until the normalization of the FS. Before the administration of the treatments, all the calves were daily submitted to a complete physical examination and FS evaluation until the resolution of diarrhea. Physical examination included evaluation from a distance plus hands-on examination, focusing on: physical appearance, body weight, body condition, head, mouth, eyes, ears, neck and back, thorax, abdomen, umbilicus, musculoskeletal system, perianal region, body temperature, feces, urine, and external genitalia [25, 26]. Dehydration status was assessed with a score system [27]. Milk intake was recorded during the entire diarrheic episode.

At the conclusion of the study, the female calves were raised in the farm as rearing cows, while the male calves were sold for fattening as meat animals.

Data concerning the weight at birth and at the third week of life, the age of diarrhea onset and the T0 fecal scores (T0-FS) recorded for both groups were assessed for normal distribution by the Shapiro-Wilk normality test and then a Mann-Whitney test was applied in order to verify differences between the two groups at the inclusion time [28].

The average daily gain between birth date and third week of life was calculated for all the calves in both groups. Data concerning the average daily gain, the DDE and fecal scores recorded throughout the DDE were analyzed by a linear model including treatments and sex and their interaction as fixed factors. A Shapiro-Wilk normality test was performed to assess data distribution. A Mann-Whitney test was carried out to verify differences between the two groups regarding the average daily gain, the DDE and the fecal scoring [28]. Values with *P* < 0.05 were considered statistically significant.

## Results

Twenty-four out of the 40 calves, 12 males and 12 females met the inclusion criteria, and were thus included in this study. The average weight at birth was 41 kg (minimum value of 34.5 kg and maximum value of 44 kg) and 37.5 kg (minimum value of 35 kg and maximum value of 48.5 kg) for groups C and T, respectively. The mean age at T0 was 7.9 ± 4.8 days for group C, and 7.2 ± 2.9 days for group T. The mean T0-FS were 1.6 ± 0.5 and 2.0 ± 0.7 for groups C and T, respectively. No significant differences were found in terms of the weight at birth, the age of diarrhea onset, and the T0-FS between the two groups (*P* > 0.05).

There were no alterations at the physical examination, and the hydration status was normal for all calves during the entire study period. No differences between groups were found for milk intake. Thus, no pharmacological treatments or fluid therapy were needed.

Seven out of twelve (7/12) calves belonging to group C resulted positive for *Cryptosporidium parvum*, 2/12 for *Rotavirus* and *Coronavirus,* and 3/12 negative. Nine/12 calves belonging to group T were positive to *Cryptosporidium parvum*, 2/12 for *Rotavirus* and *Coronavirus,* and 1/12 were negative to the rapid ELISA test performed. Fecal flotation did not show intestinal parasites.

The average weight at the third week of life was 53 kg (minimum value of 42 kg and maximum value of 64 kg) and 46.7 kg (minimum value of 46 kg and maximum value of 52.5 kg) for groups C and T, respectively. The average daily gain was 0.643 kg/day (minimum value of 0.143 kg/day and maximum value of 1.024 kg/day), and 0.442 kg/day (minimum value of 0.190 kg/day and maximum value of 0.524 kg/day), for groups C and T, respectively. Differences between the two groups were not significant.

The mean DDE in days was 10.3 ± 3.5 for group C, and 6.4 ± 3.9 for group T (Table 1). A significant difference between the two groups was found (*P* = 0.01). The mean fecal score recorded throughout the DDE for group C was 1.6 ± 0.5, while for group T the score was 1.4 ± 0.8, with a statistically significant difference between the two groups (*P* = 0.03). No statistically significant differences were found between male and female calves for the DDE and fecal score recorded throughout the DDE. No calves refused the tannins solution.

**Table 1:** Duration of the diarrheic episode (DDE), fecal score recorded throughout the DDE of control group (C) and tannin-treated group (T) expressed as mean (X) ± standard deviation (SD)

	Group C (X ± DS)	Group T (X ± DS)	*P* value
DDE (days)	10.3 ± 3.5	6.4 ± 3.9	0.0184
Fecal score recorded throughout the DDE	1.6 ± 0.5	1.4 ± 0.8	0.618

## Discussion

Due to the high amount of antimicrobials used every year in the livestock industry and possible cross-resistance between human and animal pathogens [29, 30], for many years alternative treatments have been investigated [11].

Chestnut tannins have been proposed to modulate rumen fermentation and the degradability of food proteins in cattle [16, 17]. However, there is little information on the effects of chestnut tannins on diarrhea in neonatal calves, especially in relation to different pathogens.

The pathogen that was most isolated in our population of diarrheic calves was *Cryptosporidium parvum*, in line with those reported in the literature [1, 2, 11]. The mean age of diarrhea onset for all calves included was also in line with literature data [1, 2].

It is well known that natural plant tannins are able to control intestinal parasites in ruminants [15] and, more in general, have antimicrobial properties [31]. The supplementation of chestnut extract in grazing heifers has been shown to lead to a significant increase in the average daily gain due to a decrease in fecal parasites infections, above all nematodes [32]. Several mechanisms of actions have been proposed for tannins, including enzyme inhibition and substrate or metal ions deprivation, and bacterial cell membrane integrity [31]. However, most studies have reported the effects of tannins (above all condensed tannins) on bacteria, fungi and nematode, whereas only few have investigated the effect of tannins on protozoa and, in particular, on *Cryptosporidium parvum* [11, 33].

The anti-protozoal activity of polyphenols has been reported for *Eimeria* [34–36]. A suggested mechanism is the ability of tannins to directly decrease the viability of the larval stage and disrupt egg hatching, as previously observed also for nematodes [15].

However, there have been conflicting results. Bhatta et al. (2009) found a decrease in protozoa when hydrolysable tannins from six different plant sources were applied [37]. Similar results were obtained by Tan et al. (2011), Benchaar et al. (2008) and Carulla et al. (2005) who reported that condensed tannins from *Leucaena*, quebracho and from *Acacia mearnsii* respectively, can decrease protozoal numbers [38–40]. In contrast, Vasta et al. (2010) found that quebracho tannins were able to increase protozoa in rumen liquor [41].

Differences in the concentration of tannins, plant sources, protozoal species and environment considered in such studies may explain the conflicting results obtained, because these parameters play an important role in the antiprotozoal activity of polyphenols.

To the best of our knowledge this is the first study that has investigated orally administrating tannins as a treatment for calf diarrhea. Tannins solution appeared to be pleasant for all the calves enrolled in the study and was easy to administer. The length of diarrhea was almost four days shorter in the group treated with tannins compared to the control group. Chestnut tannins seem to shorten the length of diarrhea in calves, as already reported in humans [19]. Chestnut polyphenols are ellagitannins and those from wood distillation are particularly rich in ellagic acid, such as pomegranate polyphenols [42].

Few studies have reported on the absorption and metabolism of ellagitannins in animal models. Studies of rat intestinal content demonstrated that at the caecum level ellagitannins are hydrolyzed to ellagic acid [43]. Other authors have detected free ellagic acid in human plasma after 1 h post ingestion of pomegranate and attributed this to the release after hydrolysis of ellagitannins according to an optimal physiological pH and gut microbiota activity. In addition, Cerdà et al. (2003; 2004; 2005) suggested a microbial involvement at the colon level in converting ellagic acid into urolithins, which are potent anti-inflammatory metabolites [44–46].

Although the length of diarrhea was significantly shorter in calves from group T, no significant difference was found in terms of average daily gain between control calves and calves that received chestnut tannins. Similarly, a study based on tannins from pomegranate reported no effects of tannins on average daily gain in the first 60 days of life in dairy calves [11].

Further studies are needed in order to investigate the possible effect of hydrolysable tannins on specific etiological pathogens, in particular on *Cryptosporidium parvum*, as already reported for the extract of pomegranate polyphenols [11]. Also, increasing the number of animals included might clarify the effect of chestnut tannins on the average daily gain in calves affected by diarrhea.

## Conclusions

Chestnut tannins might represent a low-impact treatment of neonatal diarrhea in calves. However, the effects of chestnut tannins on the onset of diarrhea and on the weight gain of calves need further investigation due to the lack of data on the metabolism of ellagitannins in ruminants.

## Abbreviations

C: control group
DDE: duration of a diarrheic episode
ELISA: Enzyme-Linked Immunosorbent Assay
FS: fecal score
T: tannin-treated group
